# *N*-Acetylmuramic Acid (MurNAc) Auxotrophy of the Oral Pathogen *Tannerella forsythia*: Characterization of a MurNAc Kinase and Analysis of Its Role in Cell Wall Metabolism

**DOI:** 10.3389/fmicb.2018.00019

**Published:** 2018-01-26

**Authors:** Isabel Hottmann, Valentina M. T. Mayer, Markus B. Tomek, Valentin Friedrich, Matthew B. Calvert, Alexander Titz, Christina Schäffer, Christoph Mayer

**Affiliations:** ^1^Microbiology and Biotechnology, Interfaculty Institute of Microbiology and Infection Medicine Tübingen, Department of Biology, Eberhard Karls Universität Tübingen, Tübingen, Germany; ^2^NanoGlycobiology Unit, Department of NanoBiotechnology, Universität für Bodenkultur Wien, Vienna, Austria; ^3^Chemical Biology of Carbohydrates, Helmholtz Institute for Pharmaceutical Research Saarland, Saarbrücken, Germany; ^4^Deutsches Zentrum für Infektionsforschung, Partner Site Hannover-Braunschweig, Brunswick, Germany; ^5^Department of Pharmacy, Saarland University, Saarbrücken, Germany

**Keywords:** oral pathogen, red complex consortium, *N*-acetylmuramic acid kinase, MurNAc auxotrophy, peptidoglycan metabolism, cell wall recycling

## Abstract

*Tannerella forsythia* is an anaerobic, Gram-negative oral pathogen that thrives in multispecies gingival biofilms associated with periodontitis. The bacterium is auxotrophic for the commonly essential bacterial cell wall sugar *N-*acetylmuramic acid (MurNAc) and, thus, strictly depends on an exogenous supply of MurNAc for growth and maintenance of cell morphology. A MurNAc transporter (Tf_MurT; Tanf_08375) and an ortholog of the *Escherichia coli* etherase MurQ (Tf_MurQ; Tanf_08385) converting MurNAc-6-phosphate to GlcNAc-6-phosphate were recently described for *T. forsythia.* In between the respective genes on the *T. forsythia* genome, a putative kinase gene is located. In this study, the putative kinase (Tf_MurK; Tanf_08380) was produced as a recombinant protein and biochemically characterized. Kinetic studies revealed Tf_MurK to be a 6-kinase with stringent substrate specificity for MurNAc exhibiting a 6 × 10^4^-fold higher catalytic efficiency (*k*_cat_/*K_m_*) for MurNAc than for *N-*acetylglucosamine (GlcNAc) with *k_cat_* values of 10.5 s^-1^ and 0.1 s^-1^ and *K_m_* values of 200 μM and 116 mM, respectively. The enzyme kinetic data suggest that Tf_MurK is subject to substrate inhibition (*K_i[S]_* = 4.2 mM). To assess the role of Tf_MurK in the cell wall metabolism of *T. forsythia*, a kinase deletion mutant (*ΔTf_murK::erm*) was constructed. This mutant accumulated MurNAc intracellularly in the exponential phase, indicating the capability to take up MurNAc, but inability to catabolize MurNAc. In the stationary phase, the MurNAc level was reduced in the mutant, while the level of the peptidoglycan precursor UDP-MurNAc-pentapeptide was highly elevated. Further, according to scanning electron microscopy evidence, the *ΔTf_murK::erm* mutant was more tolerant toward low MurNAc concentration in the medium (below 0.5 μg/ml) before transition from healthy, rod-shaped to fusiform cells occurred, while the parent strain required > 1 μg/ml MurNAc for optimal growth. These data reveal that *T. forsythia* readily catabolizes exogenous MurNAc but simultaneously channels a proportion of the sugar into peptidoglycan biosynthesis. Deletion of *Tf_murK* blocks MurNAc catabolism and allows the direction of MurNAc solely to peptidoglycan biosynthesis, resulting in a growth advantage in MurNAc-depleted medium. This work increases our understanding of the *T. forsythia* cell wall metabolism and may pave new routes for lead finding in the treatment of periodontitis.

## Introduction

*Tannerella forsythia* is an anaerobic, Gram-negative oral pathogen affiliated to the *Bacteroidetes* phylum of bacteria (Tanner and Izard, 2006). It acts as a late colonizer within oral biofilms and is found alongside *Porphyromonas gingivalis* and *Treponema denticola*, together constituting the so called “red complex." This bacterial consortium is associated with severe forms of periodontitis, an inflammatory oral disease of global importance that is characterized by destruction of alveolar bone and soft tissues, ultimately leading to tooth loss if untreated (Holt and Ebersole, 2005; Hajishengallis and Lamont, 2012). Anne Tanner initially described the “fusiform“ (spindle-shaped) morphology of a slow-growing *Bacteroidetes* strain isolated from the human oral cavity, formerly named *Bacteroidetes forsythus* or *Tannerella forsythensis*, and finally renamed *Tannerella forsythia* (Tanner et al., 1986). It was later recognized by Wyss that the organism has a strict dependency on the amino sugar *N*-acetylmuramic acid (MurNAc) and that growth defects and morphological changes, such as fusiform morphology, are consequences of impaired cell wall metabolism caused by MurNAc depletion (Wyss, 1989). MurNAc and *N*-acetylglucosamine (GlcNAc) are essential components of the peptidoglycan (PGN) of the bacterial cell wall. Alternatingly connected, these amino sugars form the glycan strands of PGN which are crosslinked via peptides to form a net-like polymeric fabric surrounding and stabilizing the bacterial cell and conferring cell shape (Höltje, 1998; Young, 2003).

Inspection of available *T. forsythia* genome sequences revealed that this bacterium lacks genes commonly required for the *de novo* biosynthesis of PGN in bacteria (Friedrich et al., 2015). These are the *glmS* and *glmU* genes, required for UDP-GlcNAc biosynthesis and the *murA* and *murB* genes, which encode enzymes involved in the formation of the PGN precursor uridine diphosphate-*N*-acetylmuramic acid (UDP-MurNAc) (Mengin-Lecreulx et al., 1982; Typas et al., 2012). *T. forsythia’s* inability to *de novo* synthesize MurNAc implicates that the bacterium has to attain this compound from external sources for viability. *Escherichia coli* and other bacteria possess a phosphotransferase system (PTS) transporter (MurP) for the uptake and concomitant phosphorylation of MurNAc yielding MurNAc-6P (Dahl et al., 2004; Borisova et al., 2016), as well as a MurNAc-6P etherase (MurQ) for catabolization of MurNAc-6P by cleaving off the lactyl ether substituent, yielding GlcNAc-6P and D-lactate (Jaeger et al., 2005; Hadi et al., 2008; Jaeger and Mayer, 2008). According to genome analysis, in *T. forsythia*, PTS-type transporters are missing; however, recently, a PTS-independent uptake system for MurNAc (Tf_MurT; Tanf_08375) was identified in the *T. forsythia* type strain ATCC 43037, which belongs to the sodium symporter superfamily (Ruscitto et al., 2016). The corresponding *Tf_murT* gene is present within an operon, together with an ortholog of the *E. coli* MurNAc-6-phosphate etherase gene *murQ* (*Tf_murQ; Tanf_08385*) and a putative sugar kinase gene *Tf_murK* (*Tanf_08380)*. We have shown in a recent study, that an *E. coli* MurNAc-PTS transporter mutant (*ΔmurP*) can be rescued for growth on MurNAc as sole carbon source only upon co-expression of the transporter Tf_MurT and the putative kinase Tf_MurK. This suggested that Tf_MurK would phosphorylate MurNAc, yielding MurNAc-6P, which would be subsequently cleaved by the etherase Tf_MurQ, yielding GlcNAc-6P (Ruscitto et al., 2016).

In the present study, we biochemically characterized the *T. forsythia* kinase Tf_MurK of the *Tf_murTKQ* operon from the type strain ATCC 43037 revealing stringent specificity of the enzyme for MurNAc. Further, we constructed a *Tf_murK* deletion mutant and characterized changes of this mutant in cell wall metabolism in comparison to the parental strain, providing evidence for the steady uptake of exogenous MurNAc by *T. forsythia* cells as well as for the presence of a novel pathway that channels MurNAc to PGN biosynthesis and is elaborated in parallel to MurNAc catabolism.

## Materials and Methods

### Bacterial Strains, Growth Conditions, and Growth Curves

*T. forsythia* type stain ATCC 43037 - in the following referred to as *T. forsythia* wild-type (WT) - was obtained from the American Type Culture Collection (Manassas, VA, United States). *T. forsythia* WT and an isogenic Δ*Tf_murK::erm* mutant created in the course of this study (see Supplementary Figure S1) were grown in liquid or solid-agar brain heart infusion (BHI) medium (37 g/l; Oxoid, Basingstoke, United Kingdom) at 37° C for 4–7 days under anaerobic conditions in an anaerobe jar (AnaeroJar; Oxoid). The media were supplemented with 10 g/l yeast extract (Sigma, Vienna, Austria), 1 g/l L-cysteine (Sigma), 5 μg/ml hemine (Sigma), 2 μg/ml menadione (Sigma), 5%(v/v) horse serum (Thermo Fisher Scientific, Vienna, Austria), and MurNAc (Carbosynth, Compton, United Kingdom) at a concentration of 20 μg/ml (Tomek et al., 2014) if not stated otherwise. Erythromycin (Erm; 5 μg/ml) and gentamycin (Gm; 50 μg/ml) were added to the media when appropriate.

To determine the influence of MurNAc depletion on the growth of *T. forsythia* WT and Δ*Tf_murK::erm* mutant, growth curves were recorded upon supplementation of the culture medium with 0.1, 0.5, 1.0, and 20.0 μg/ml MurNAc. Bacterial cells were inoculated to a starting optical density at 600 nm (OD_600_) of 0.1 and grown until the stationary phase had been reached (75 h). Biological triplicates were measured with a cell density meter (Ultraspec 10; Amersham Biosciences, Austria), three times, each, at any given time point.

*Escherichia coli* BL21(DE3) was grown in lysogeny broth (LB Lennox, 10 g/l tryptone, 5 g/l yeast extract, 5 g/l NaCl) at 37° C under continuous shaking at 140 rpm; kanamycin (Km; 50 μg/ml) was added when appropriate.

### Plasmid Construction, Expression, and Purification of Tf_MurK

For recombinant production of C-terminally His_6_-tagged Tf_MurK in *E. coli*, the *T. forsythia murK* gene (*Tf_murK*, *Tanf_08380*) was cloned in the expression vector pET28a (Novagen, Darmstadt, Germany). Genomic DNA of *T. forsythia* ATCC 43037 was prepared, using the GenElute Bacterial Genomic DNA Kit (Sigma, Vienna, Austria) and served as a template to amplify a 852-bp DNA fragment containing *Tf_murK* by PCR, using the primer pair 1068for/1068rev (**Table 1**). PCR product and vector were digested with *Nco*I and *Xho*I (NEB, Frankfurt, Germany) and ligated into pET28a using T4 DNA ligase (Thermo Fisher Scientific, Waltham, MA, United States). The resulting plasmid, was named pET28_Tf_MurK and transformed into *E. coli* BL21(DE3) by electroporation.

**Table 1:** Oligonucleotide primers used for PCR amplification reactions.

Primer	Sequence (5′-3′)
1068for	GCG*CCATGG*CGATACTGATTGCAGATAGC
1068rev	GCG*CTCGAG*TACGGTTTTTGCAACTGTCGAATAG
622	ATAATCCCGGATCATGGTCGTTCG
623	CTTTGCGCACCCGACGAGATGATG
624	TTCAGACGCCGGAAGAGATG
625	GGATTGCGAACGATTGTACC
1068upfor	ACACCGACCGACCTCGTATTTCCTTTC
1068uprev	**CGAACGGGCAATTTCTTTTTTGTCAT**ATTTTGATATATATTTTTTTCTTATACAAGAT
1068downfor	**GTCCCTGAAAAATTTCATCCTTCGTAG**ATGACATTTATCAAAATAACAGAACAGG
1068downrev	GGTAAGCGGTCATCATCTCTCGTCGG
524	GTAAAACGAACGGGCAATTTCTTTTTTGTCAT
525	CCCTGAAAAATTTCATCCTTCGTAG
460	ATGACAAAAAAGAAATTGCCCGTTCGTTTTAC
461	CTACGAAGGATGAAATTTTTCAGGGACAAC

*Nucleotides used for overlap-extension PCRs are written in bold, artificially introduced restriction enzyme sites are italicized.*

For overexpression of Tf_MurK, 2 l of LB medium supplemented with Km were inoculated with 20 ml of an overnight culture of *E. coli* BL21(DE3) harboring pET28_Tf_MurK. The cells were grown at 37° C in a baffled 5 l–flask and vigorous shaking. At an OD_600_ ∼0.7, protein expression was induced by addition of isopropyl-β-D-thiogalactopyranoside (IPTG; 1 mM final concentration) and the culture was further incubated overnight at 20° C. Cells were harvested by centrifugation at 4000 *g* for 20 min at 4° C (F12-6 x 500 LEX rotor, Thermo Fisher Scientific, Waltham, MA, United States). The cell pellet was resuspended in 20 ml of 20 mM Na_2_HPO_4_ (pH 7.5) containing 500 mM NaCl and 1 mM DTT (buffer A), and cell lysis was achieved using a French cell disruptor (Sim Aminco Spectronic Instruments, Inc. Rochester, NY, United States), three times at 1’000 psi. Subsequently, the soluble extract was separated from cell debris by centrifugation at 38’000 *g* (Sorvall, SS-34 rotor, Beckmann, Krefeld, Germany) for 60 min at 4° C.

For purification of recombinant Tf_MurK (rTf_MurK) by Ni^2+^ affinity chromatography, the supernatant obtained before was filtered through a 0.2-μm filter (Sarstedt, Nümbrecht, Germany) and loaded on a 1-ml His-Trap column (GE Healthcare, Freiburg, Germany), pre-equilibrated with ten column volumes each of H_2_O and buffer A (20 mM Na_2_HPO_4_, 500 mM NaCl, 1 mM DTT, pH 7.5,), using a protein purification system (Äkta Purifier, GE Healthcare). Protein elution was achieved by applying a linear gradient from 0 to 500 mM imidazole in buffer A. Elution fractions were analyzed by SDS-PAGE using 12% polyacrylamide gels stained with Coomassie Brilliant Blue G250 (Laemmli, 1970) and rTf_MurK-containing fractions were pooled and applied to size-exclusion-chromatography (HiLoad 16/60 Superdex 200 column, GE Healthcare) using buffer A as eluent; fractions containing pure rTf_MurK according to SDS-PAGE were pooled. The protein concentration of the rTf_MurK pool was calculated using the extinction coefficient at 280 nm (20,775 M^-1^ cm^-1^, ExPASy, ProtParam tool) as measured in a 1-ml quartz cuvette (Hellma, Müllheim, Germany) using a SpectraMax M2 spectrometer (Molecular Devices, Biberach, Germany).

### Activity of rTF_MurK, Mg^2+^-Dependency and Identification of the Reaction Product

Product formation upon rTf_MurK kinase activity was analyzed by electrospray ionization-time of flight mass spectrometry (ESI-TOF-MS) using a MicrO-TOF II (Bruker Daltonics, Bremen Germany), operated in negative ion mode, after separation on an UltiMate 3000 RS, high-performance liquid chromatography (HPLC) system (Dionex, Thermo Scientific, Sunnyvale, USA). 1 μg of Tf_MurK was added to a 100-μl reaction mixture containing 1 mM MurNAc, 10 mM ATP, 100 mM Tris-HCl (pH 7.6) and incubated for 1 h at room temperature (RT). In a parallel approach, 10 mM MgCl_2_ was added to the reaction mixture to determine the effect of Mg^2+^ on catalysis. Reaction aliquots of 10 μl, each, were separated on a ZIC-HILIC column (150 × 7.5 mm, 200 Å, 5 μm; Merck) at 37° C, applying a 40-min elution program as described previously (Unsleber et al., 2017). Extracted ion chromatograms (EICs) for MurNAc-P [(M-H)^-1^= 372.070 *m/z*] were obtained with the software Data Analysis (Bruker), the area under the curve (AUC) values for the EICs for MurNAc-P were determined using the Prism 6 program (GraphPad Software, La Jolla, CA, United States) and the relative activity of the enzyme was calculated using these values following a published protocol (Borisova and Mayer, 2017).

The *m/z* value obtained by MS analyses does not allow to distinguish between the stereochemistry of phosphosugars. To show that the product formed by Tf_MurK is indeed MurNAc-6P, it was cleaved with the etherase rTf_MurQ - available in our laboratory from a previous study (Ruscitto et al., 2016) - which specifically converts this phosphosugar into GlcNAc-6P and D-lactate. For this assay, 1 μg of rTf_MurK was added to a 100-μl reaction mixture containing 5 mM MurNAc, 10 mM ATP and 10 mM MgCl_2_ in 100 mM Tris-HCl (pH 7.6) and incubated overnight at RT. Subsequently, 10 μg (2 μl) of rTf_MurQ were mixed with 20 μl of the reaction and incubated for 1 h at 37° C. The negative control contained 2 μl of distilled water instead of rTf_MurQ. After stopping the reaction, the product was analyzed by ESI-TOF-MS as described above except for using a Gemini C18 HPLC column (150 × 4.6 mm, 110 Å, 5 μm; Phenomenex, Aschaffenburg, Germany) (Borisova et al., 2014).

### Determination of the Substrate Specificity of rTf_MurK

Substrate specificity of rTf_MurK was assayed as described previously (Reith et al., 2011). ATP, glucose (Glc), GlcNAc, glucosamine (GlcN), *N*-acetyl galactosamine (GalNAc) and GlcNAc-6P were obtained from Sigma–Aldrich (Taufkirchen, Germany), MurNAc was obtained from Bachem (Bubendorf, Switzerland). MurNAc-6P was available from a previous study (Unsleber et al., 2017) and 1,6-anhydro-MurNAc (anhMurNAc) was synthesized according to a published protocol (Calvert et al., 2017).

For the substrate specificity assay, the different sugars - MurNAc, GlcNAc, GalNAc or GlcN at 50 mM final concentration, each, Glc at 1 mM final concentration or anhMurNAc at 10 mM final concentration - were added to a 100-μl reaction mixture containing 100 mM Tris-HCl (pH 7.6), 100 mM ATP and 10 mM MgCl_2_. The reaction was started by addition of rTf_MurK (10 nM final concentration) and continued for 16 h at RT. 3-μl samples were taken from each reaction mixture at time points 0 (t_0_) and 16 h (t_16_) and spotted on a TLC plate (Silica 60 F_254_ Merck, Darmstadt, Germany). The reaction mixtures were separated using a basic solvent of *n*-butyl alcohol/methanol/25% (w/v) ammonium hydroxide/water in a ratio of 5:4:2:1 (v/v/v/v). The separated compounds were visualized by carbonization, for which the TLC plate was quickly dipped in a 5% methanolic solution of sulfuric acid, followed by drying and final development of the plate by heating for 15 min at 180° C.

### Determination of pH Optimum and Temperature Stability of Tf_MurK

To determine pH stability and pH optimum of rTf_MurK, buffers in the pH range of 2.0–11.0 were used, i.e., Clark and Lubs buffer (pH 2.0), sodium acetate buffer (pH 3.0–6.0), sodium phosphate buffer (pH 6.0–8.0), and sodium carbonate buffer (pH 9.0–11.0). For the pH stability test, rTf_MurK was diluted in buffer to a final concentration of 2 ng/ml and pre-incubated for 30 min at 20° C. The reaction was started by adding 5 μl of the pre-incubated enzyme (10 ng) to a 45-μl mixture containing 1 mM MurNAc, 10 mM MgCl_2_ and 10 mM ATP in 50 mM phosphate buffer (pH 7.0). After incubation for 30 min at 20° C, the reaction was stopped by adding 50 μl of a solution containing 1% formic acid and 0.5% ammonium formate (pH 3.2; stopping solution). For the pH optimum test, 100-μl reaction mixtures were prepared, containing 1 mM MurNAc, 10 mM MgCl_2_ and 10 mM ATP, in a 50 mM buffer of a particular pH in the range of 2.0 to 11.0. The reaction was started by adding 10 ng of rTf_MurK followed by incubation for 30 min at 20° C; the reaction was stopped by adding 100 μl of stopping solution. Samples were analyzed by HPLC connected to ESI-TOF-MS (MicrO-TOF II; Bruker) and quantified using the Prism 6 program (GraphPad), as described above (Borisova and Mayer, 2017;Unsleber et al., 2017).

To determine the temperature stability of rTf_MurK, the purified enzyme was pre-incubated at different temperatures (i.e., 4, 20, 37, 45, 55, and 65° C) for 30 min. Subsequently, an aliquot of that solution corresponding to 10 ng of rTf_MurK was added to a 50 μl reaction mixture (20° C). The reaction was carried out and terminated as described above. To investigate the temperature optimum of rTf_MurK, a standard 50-μl reaction containing 10 ng of rTf_MurK was carried out for 30 min at different temperatures (i.e., 4, 20; 37, 45, 55, and 65° C) and samples were subsequently analyzed and the reaction quantified as described above.

### Determination of Enzyme Kinetic Parameters

Kinetic parameters of Tf_MurK-catalyzed phosphorylation of MurNAc and GlcNAc with ATP were determined by using a coupled enzyme assay as described previously (Reith et al., 2011), with minor modifications. In a 96-well plate (Greiner, Frickenhausen, Germany), a 100-μl reaction mixture containing additionally 1 mM phosphoenolpyruvate, 0.2 mM NADH, 10 U of pyruvate kinase, and 7 U of lactate dehydrogenase (all from Sigma–Aldrich, Taufkirchen, Germany) was incubated with the amino sugar substrates, ranging from 0.05 to 2 mM for MurNAc or 0.1 to 250 mM for GlcNAc. The reaction was started by the addition of freshly prepared rTf_MurK; 10 ng (3 nmol) enzyme was used for the reaction with MurNAc and 1 μg (300 nmol) enzyme for the reaction with GlcNAc. The change of NADH absorbance was monitored at 340 nm in a spectrophotometer (Spark 10 M; Tecan, Männedorf, Switzerland) for 45 min at 20° C. The experimental data were fitted to the Michaelis-Menten equation and, taking into account substrate inhibition, also to the equation Y = v_max_^∗^[S]/(K_m_ + [S]^∗^(1+[S]/Ki), using the program GraphPad Prism 6. The molar extinction coefficient of NADH at 340 nm (6220 M^-1^ cm^-1^) was used to calculate *v*_max_ and *k*_cat_ values.

### Construction of a *T. forsythia* MurNAc 6-Kinase Deletion Mutant

A knock-out vector was constructed to exchange the *Tf_murK* gene of *T. forsythia* ATCC 43037 (*Tanf_08380*) in frame with an erythromycin resistance (*erm*) marker (see Supplementary Figure S1). A detailed description of the cloning procedure and the transformation of the knock-out cassettes into *T. forsythia* is published elsewhere (Tomek et al., 2014, 2017). For PCR amplifications, Phusion High-Fidelity DNA polymerase (Thermo Fisher Scientific, Austria) was used according to the manufacturer’s instructions. Oligonucleotides (Thermo Fisher Scientific) used in this study are listed in **Table 1**. Extraction of genomic DNA was conducted according to a published protocol (Cheng and Jiang, 2006). The knock-out vector contains two homology regions approximately 1-kbp up-stream and down-stream of *Tf_murK* and the *erm* marker cloned in between. Primer pairs 1068upfor/1068uprev and 1068downfor/1068downrev, respectively, were used to amplify the up- and down-stream homology regions from genomic DNA of *T. forsythia* ATCC 43037. The *erm* gene (805 bp, without the promotor region) of pJET/TF0955ko (Tomek et al., 2014) was amplified using primers 460 and 461. Subsequently, the knock-out cassette was blunt-end cloned into the cloning vector pJET1.2, creating the final knock-out vector pJET1.2/Δ*Tanf_08380*. Transformed and viable clones on selective plates containing Erm were further tested for correct integration of the knock-out cassette by screening PCR and sequencing (Supplementary Figure S1). The deletion mutant strain, thus, carries an *erm* marker in place of the *Tf_murK* gene and, accordingly, was named Δ*Tf_murK::erm*.

### Preparation of Cytosolic Fractions and Metabolite Analysis

The intracellular accumulation of cell wall metabolites in *T. forsythia* Δ*Tf_murK::erm* in comparison to *T. forsythia* WT was investigated in both the exponential and the stationary phase; these phases had been determined before by recording growth curves of the strains as described above (time points are indicated in **Figure 4**). For that purpose, two different volumes of liquid medium (i.e., 50 and 200 ml) supplemented with 20 μg/ml MurNAc (the MurNAc concentration routinely used for optimal growth of the bacterium) and appropriate antibiotics were inoculated with *T. forsythia* WT and mutant cells at an OD_600_ ∼0.05. The 200-ml cultures were harvested (5000 *g*) after 3 days of incubation under anaerobic conditions, corresponding to the exponential growth phase (OD_600_ ∼0.6); the 50-ml cultures were harvested on day 4, corresponding to the stationary growth phase (OD_600_ ∼1.7, wild-type; OD_600_ ∼1.2, mutant). Metabolite extraction was performed as described with minor modifications (Gisin et al., 2013; Borisova et al., 2014). After correcting for differences in ODs and culture volumes, equal amounts of cells were washed with 20 ml of 10 mM Tris-HCl (pH 8.0), resuspended in 400 μl of Millipore water, boiled at 100° C for 1 h and centrifuged at 21000 *g* for 15 min. The supernatant was then transferred into a fresh tube and treated with 1.5 ml of acetone (HPLC grade). After centrifugation at 21000 *g* for 15 min, the supernatant was transferred into a fresh tube and left open to evaporate under vacuum overnight at 37° C. The residual liquid was dried off in a centrifugal evaporator. Dried samples were resuspended in 50 μl of Millipore water and 5-μl aliquots were analyzed by LC-MS using a Gemini C18 column (150 × 4.6 mm, 110 Å, 5 μm; Phenomenex) and a UltriMate 3000 RS (Dionex) coupled to a MicrO-TOF II mass spectrometer (Bruker) operated in negative ion mode (Borisova et al., 2014). EICs were used to calculate the area under the curve (AUC) using Prism6 (GraphPad). Data were presented as mean of three replicates.

### Scanning Electron Microscopy (SEM)

*T. forsythia* WT and *ΔTf_murK*::*erm* mutant were cultivated as described above, using MurNAc concentrations of 0.1, 0.5, 1.0, and 20.0 μg/ml, respectively. Briefly, at an OD_600_ of ∼0.5 (exponential growth phase) or ∼1.5 (stationary growth phase), 1 ml of bacterial culture was harvested, each, and centrifuged at 5000 *g* for 7 min. Cell pellets were washed twice with phosphate-buffered saline (PBS), suspended in 500 μl of ethanol (25% in PBS), incubated for 7 min at RT and centrifuged. This step was repeated using solutions of 35, 50, 60, 70, 80, 90, and 95% ethanol in PBS and finally 100% ethanol. Finally, the samples were sputter-coated with gold (EM SDC005 apparatus; Leica, Wetzlar, Germany) and imaged with an Inspect S50 scanning electron microscope (FEI, Eindhoven, Netherlands). A detailed description of sample preparation for sputter-coating and SEM is published elsewhere (Tomek et al., 2014).

## Results

### Tf_MurK Is a Specific MurNAc-6-Kinase

To determine function and specificity of the putative kinase Tf_MurK (Tanf_08380), the 283-amino acid enzyme (theoretical pI, 6.54; calculated molecular mass, 31.3 kDa) was produced as a recombinant protein (rTf_MurK) in *E. coli* BL21(DE3) cells. The protein was equipped with a C-terminal His_6_-tag for purification purposes. Applying Ni^2+^ affinity chromatography followed by size exclusion chromatography, rTf_MurK was purified to near homogeneity, as judged by SDS-PAGE analysis (Supplementary Figure S2), whereat rTf_MurK migrated at about the expected size. rTf_MurK was obtained at a yield of 4 mg per liter of bacterial culture.

**FIGURE 1:**
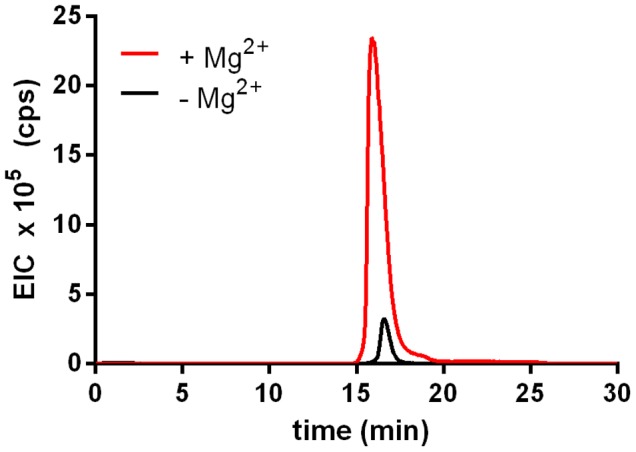
Activity of rTf_MurK is increased in the presence of magnesium ions. Product yield (MurNAc-6P) increased more than 10-fold (red) when MgCl_2_ was added (10 mM final concentration) to the Tf_MurK reaction mixture compared to the reaction without MgCl_2_ (black). Shown are the extracted ion chromatograms (EICs) for MurNAc-6P [(M-H)^-^ = 372.072 *m/z*] eluting at a retention time of 17 min on ZIC-HILIC column.

**FIGURE 2:**
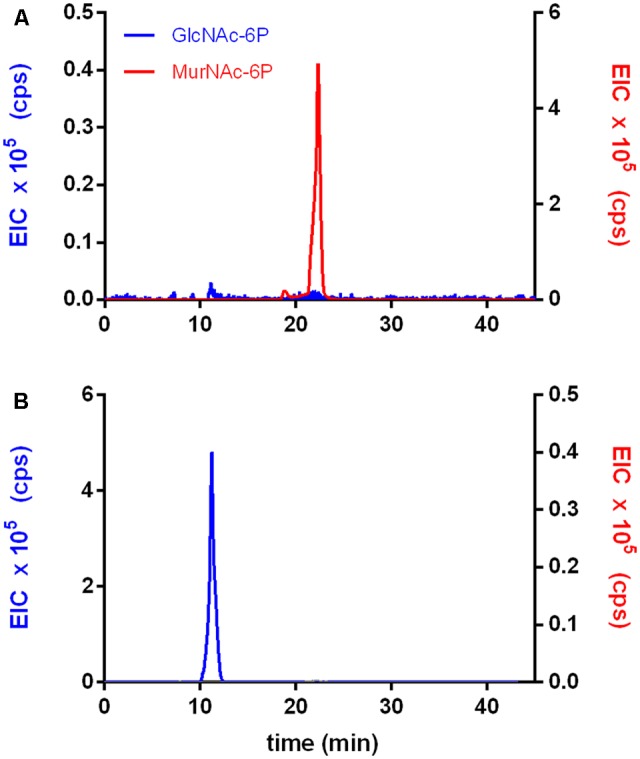
The rTf_MurK reaction product is MurNAc-6-phosphate (MurNAc-6P). The product of the rTf_MurK kinase reaction with the substrate MurNAc, MurNAc-6P (red) **(A)** was degraded by the specific MurNAc-6P etherase of *T. forsythia* (rTf_MurQ) yielding the reaction product GlcNAc-6P (blue) as followed by LC-MS **(B)**. Shown are the extracted ion chromatograms (EICs) in negative ion mode: (M-H)^-^ = 372.072 *m/z* for MurNAc-6P (red) at a retention time of 22 min and (M-H)^-^ = 300.059 *m/z* for GlcNAc-6P (blue) at a retention time of 10 min on a Gemini C-18 RP-column.

The activity of rTf_MurK was first assayed with MurNAc and ATP as substrates applying ESI-TOF-MS (**Figure 1**). A product at low intensity (EIC peak height of 2.6 × 10^5^ cps) appeared with a mass in negative ion mode (M-H)^-^ of 372.072 *m/z,* which is in agreement with the *m/z* of a supposed MurNAc-phosphate product. The product yield increased ∼10-fold (EIC peak height of 2.3 × 10^6^ cps) upon addition of 10 mM MgCl_2_ to the reaction, (**Figure 1**), indicating that MurK activity is dependent on Mg^2+^ ions. Thus, all subsequent reactions were supplemented with 10 mM MgCl_2_. We next determined the stereochemistry of the product generated upon rTf_MurK catalysis using *T. forsythia* MurNAc-6P etherase (rTf_MurQ) (Ruscitto et al., 2016). The rTf_MurK product ((M-H)^-^ = 372.072 *m/z,* retention time of 22 min) was completely converted by rTf_MurQ into a product with (M-H)^-^ = 300.059 *m/z* (retention time 12 min), which is in agreement with the expected mass of GlcNAc-6P (**Figure 2**). Hence, we identified the rTf_MurK reaction product using MurNAc and ATP as MurNAc-6P.

**FIGURE 3:**
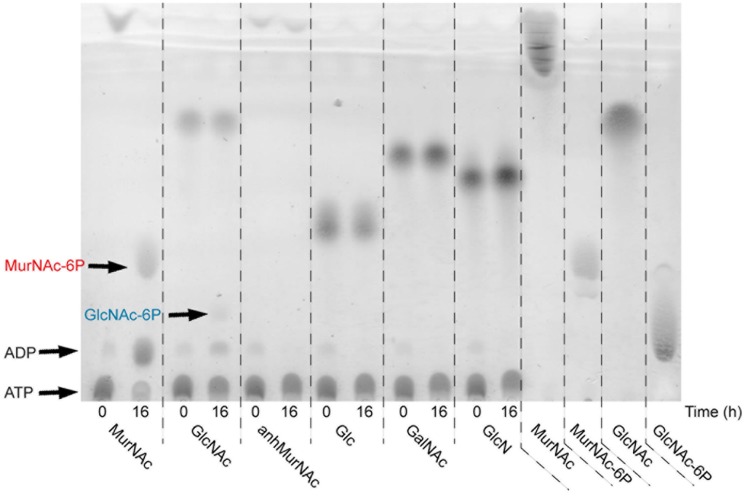
Substrate specificity of rTf_MurK analyzed by TLC. Kinase reaction mixtures containing a sugar substrate as indicated were spotted immediately (0) and after incubation for 16 h at RT (16). MurNAc, GlcNAc, MurNAc-6P, and GlcNAc-6P served as standards. ATP remains near the spotting point and the reaction product ADP slightly moves away from this point. The spots representing the reaction products MurNAc-6P (red) and GlcNAc-6P (blue) are indicated.

To determine the substrate specificity of rTf_MurK, different sugar substrates, including MurNAc, GlcNAc, anhMurNAc, Glc, GalNAc, and GlcN, were tested in a 16-h reaction followed by TLC analysis (**Figure 3**). Of the sugars tested, only MurNAc and GlcNAc, albeit apparently every slow, were converted by rTf_MurK to the corresponding phosphosugar; simultaneously ATP was converted into ADP, which was also detected on the TLC plate. The other tested sugars, including anhMurNAc, Glc, GalNAc, and GlcN obviously did not serve as substrates for the rTf_MurK reaction (**Figure 3**).

### Biochemical Characterization of Tf_MurK and Determination of Kinetic Parameters

Prior to the determination of the kinetic parameters, the pH and temperature optima of rTf_MurK were determined. Product formation of the enzyme with MurNAc and ATP was followed at different pH values and temperatures by determining the EICs of MurNAc-6P ((M-H)^-^= 372.070 *m/z*) and quantifying the AUC in comparison to a standard. rTf_MurK was shown to be stable over a wide pH range between 3.0 and 11.0, with maximal activity detected between pH 7.0 and 9.0 (Supplementary Figure S3). For determining enzyme kinetic parameters, 50 mM phosphate buffer (pH 7.0) was chosen, because the enzyme’s activity was highest in that buffer; furthermore, the buffering capacity is maximal in the optimal pH range of the enzyme. The temperature optimum for the rTf_MurK reaction was determined to be 37° C, when the reaction time was restricted to 3 min (Supplementary Figure S3). However, incubation at 37° C for 30 min almost completely inactivated the enzyme and incubation at 20° C for 30 min reduced the activity by ∼50% (Supplementary Figure S3). We thus limited the reaction time to 3 min in the kinetic experiments and choose a reaction temperature of 20° C, as a compromise between sufficient activity and stability of rTf_MurK.

For the determination of rTf_MurK kinetic parameters we used a coupled enzyme assay (Reith et al., 2011) in which the formation of ADP is stoichiometrically coupled to NADH oxidation by pyruvate kinase and lactate dehydrogenase. Kinetic parameters were calculated therefrom (**Table 2**; see also Supplementary Figure S4). The reaction of rTf_MurK with MurNAc as substrate was much faster than that with GlcNAc as substrate (*V*_max_ of 39.5 *versus* 0.5 μmol/min mg) and a 1000-fold lower *K_m_* was determined for MurNAc compared to GlcNAc (*K_m_* of 113 *versus* 116700 μM). With the latter substrate, the maximum activity at saturation was not reached, not even with 250 mM GlcNAc added to the reaction. With MurNAc, saturation was reached; however, at concentrations higher than 1 mM the enzyme’s activity dropped slightly, indicating that the enzyme was subject to substrate inhibition. Thus, the kinetic parameters were re-fitted to an equation that considers substrate inhibition. This yielded kinetic parameters of rTf_MurK for MurNAc of *K_m_* of 200 μM and a *V*_max_ of 52.6 μmol/min mg (*k*_cat_ of 10.5 s^-1^), and a MurNAc inhibitory constant (*K_i[S]_*) of 4.2 mM was determined (**Table 2**).

**Table 2:** Kinetic parameters of Tf_MurK.

Substrate	***K_M_*** [μM]	***V_**max**_*** [μmol min^**-1**^ mg^**-1**^]	***k_**cat**_*** [s^**-1**^]	***k_**cat**_***/***K_M_*** [s^**-1**^ M^**-1**^]	***K_**i(S)**_*** [mM]
	**Kinetic parameters fitted to Michaelis-Menten equation:**				
MurNAc	113	39.5	7.9	69910	nd
GlcNAc	116700	0.5	0.1	0.86	nd
	**Kinetic parameters fitted considering substrate inhibition:**				
MurNAc	200	52.6	10.5	52550	4.2

*nd, not determined.*

### Growth Advantage of a *Tf_murK* Mutant in MurNAc-Limited Medium

**FIGURE 4:**
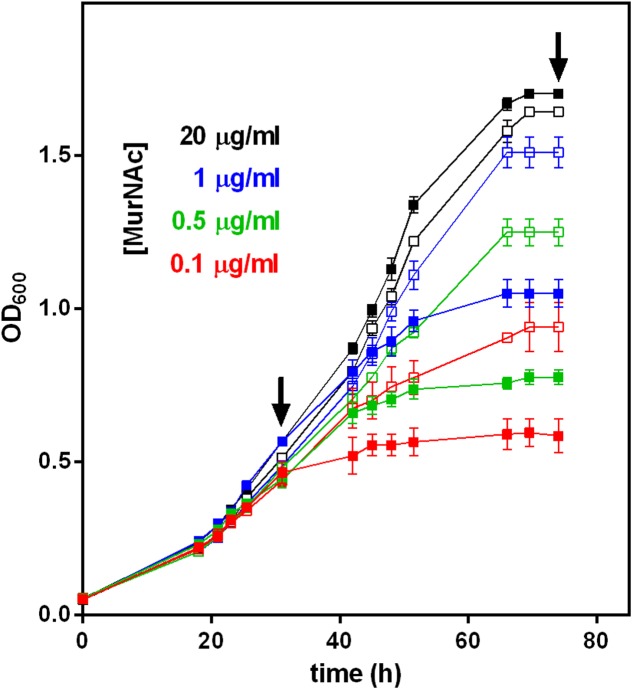
Growth of *T. forsythia* WT and *ΔTf_murK::erm* mutant in the presence of different MurNAc concentrations. The OD_600_ of cultures of *T. forsythia* WT (solid symbols) and *ΔTf_murK::erm* mutant (open symbols) was measured (shown are the mean values of triplicates and standard error) and contained MurNAc in concentrations indicated (color-coded). Arrows indicate the time points referred to as exponential and stationary phase, at which metabolite accumulation and cell morphology was analyzed (see **Figures 5**, **6**).

A *Tf_murK* mutant was constructed by insertional inactivation of the *Tanf_08380* gene using an *erm* marker (*ΔTf_murK::erm*) (Supplementary Figure S1). This strategy of mutation in *T. forsythia* has been established in our laboratory (Tomek et al., 2017). A comparison of the SDS-PAGE migration pattern of the *ΔTf_murK::erm* mutant with that of *T. forsythia* WT cells did not reveal major difference in cellular proteins; especially the presence of the two S-layer proteins TfsA and TfsB characteristic of optimally growing *T. forsythia* cells was a clear indication that no changes had occurred in the cell wall composition of the mutant (Supplementary Figure S1). In addition, when grown in complex medium supplemented with an excess of MurNAc (20 μg/ml), *T. forsythia* WT and *ΔTf_murK::erm* mutant showed very similar growth curves, reaching both a maximum of OD_600_ of ∼1.6 after 70 h (**Figure 4**).

However, when the MurNAc concentration in the medium was reduced to 1.0, 0.5, or 0.1 μg/ml, growth of WT and mutant cells was different. For the initial 30 h roughly corresponding to the exponential growth phase, *T. forsythia* WT and mutant cells grew identically in complex medium supplemented with limiting amounts of MurNAc (1.0, 0.5, or 0.1 μg/ml). After this time point, however, the mutant grew to higher OD_600_ values compared to the WT, revealing a clear growth advantage during the late exponential and stationary phases (**Figure 4**). In MurNAc-limited medium *T. forsythia* WT cells reached final OD_600_ values of ∼1.0, ∼0.8, and ∼0.6, respectively (**Figure 4**). In contrast, mutant cells continued their growth and reached under the same conditions final OD_600_ values of 1.5 (almost matching growth upon 20 μg/ml MurNAc supplementation), 1.2 and 0.9, respectively (**Figure 4**).

### Growth Phase-Dependent Accumulation of MurNAc and UDP-MurNAc- Pentapeptide in a *Tf_murK* Mutant

In an attempt to understand the unexpected growth advantage of the of *ΔTf_murK::erm* mutant under MurNAc limitation, we examined the accumulation of MurNAc and other cell wall metabolites within the cytosolic fractions of mutant and WT cells by LC-MS, comparing the situation in the exponential growth phase at 30 h (OD_600_ ∼0.5) and stationary phase at 75 h (OD_600_ ∼1.6) of growth (indicated with arrows in **Figure 4**).

**FIGURE 5:**
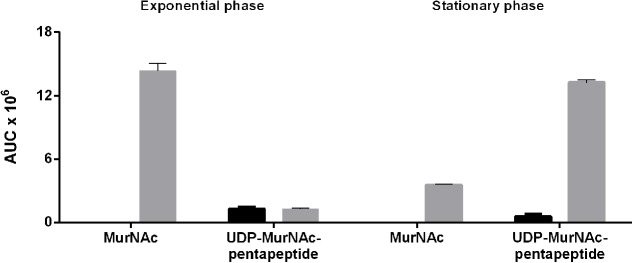
Accumulation of MurNAc and UDP-MurNAc-pentapeptide in *T. forsythia* WT and *ΔTf_murK::erm* cells in the exponential and stationary phase. Relative amounts of MurNAc and UDP-MurNAc-pentapeptide in extracts of *T. forsythia* WT (black) and *ΔTf_murK::erm* cells (gray) were determined by mass spectrometry. Shown are the mean values of three replicates calculated from the area under the curve (AUC) determined for exponential phase cells (adjusted to OD_600_ = 0.5) and stationary phase cells (adjusted to OD_600_ = 1.5).

During the exponential growth phase, the *ΔTf_murK::erm* mutant accumulated two major metabolites with *m/z* of (M-H)^-^ = 292.107 and (M-2H)^2-^ = 595.670, corresponding to MurNAc and UDP-MurNAc-pentapeptide, respectively (Supplementary Figure S5). The theoretical *m/z* values of the investigated molecules are 292.110 for MurNAc and 1192.340 for UDP-MurNAc-pentapeptide. Of the latter, the single charged ion was detected only with low intensity and mainly the doubly charged ion (M-2H)^2-^ = 595.670 *m/z* appeared. This was used to quantify the UDP-MurNAc-pentapeptide content in the extracts (Supplementary Figure S5). MurNAc appeared as a double peak, because of the separation of the α- and β-anomers (Supplementary Figure S5C). **Figure 5** summarizes the accumulation data determined by integration of the EICs that were obtained by MS measurements. Assuming that MurNAc and UDP-MurNAc-pentapeptide have roughly the same response factors in the MS experiments, we can conclude that in the exponential growth phase, the relative concentration of MurNAc in the mutant cell extract was ∼10-fold higher than that of UDP-MurNAc-pentapeptide (**Figure 5**). *T. forsythia* WT cells, in contrast, showed no accumulation of MurNAc, but yielded UDP-MurNAc-pentapeptide in the exponential growth phase, in the same relative concentration as the *ΔTf_murK::erm* mutant (**Figure 5** and Supplementary Figure S5). However, when *ΔTf_murK::erm* cells from the stationary phase were analyzed, a different accumulation pattern was observed in comparison to that from the exponential growth phase. Here, the intracellular concentration of MurNAc decreased almost 5-fold and that of UDP-MurNAc-pentapeptide increased roughly 10-fold (**Figure 5**), thus reversing the MurNAc:UDP-MurNAc-pentapeptide ratio from the exponential growth phase. For *T. forsythia* WT cells, a slight decrease of the UDP-MurNAc-pentapeptide level could be observed (**Figure 5**).

Thus, in *T. forsythia* WT cells, in neither growth phase, a measurable level of MurNAc was detected, indicating rapid metabolization. In *ΔTf_murK::erm* cells, in contrast, MurNAc and UDP-MurNAc-pentapeptide readily accumulated – with a MurNAc:UDP-MurNAc-pentapeptide ratio of 10:1 and 1:5 in the exponential and stationary growth phase, respectively.

### Morphological Defects of *T. forsythia* Cells Grown under MurNAc Limitation

**FIGURE 6:**
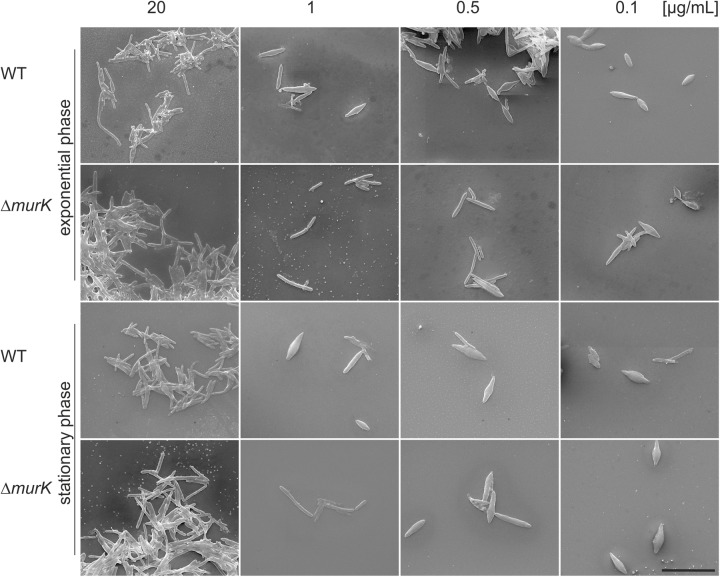
SEM micrographs of *T. forsythia* wild-type (WT) and *ΔTf_murK::erm* (*ΔmurK)* mutant cells when grown under full MurNAc supplementation (first row) and under MurNAc limiting conditions (rows two to four). Media were supplemented with 20 μg/ml (full supplementation), 1 μg/ml, 0.5 μg/ml, and 0.1 μg/ml of MurNAc. The *ΔmurK* mutant is more tolerant toward MurNAc limitation as can be seen from transition from rod-shaped to fusiform cells occurring only at 0.1 μg/ml MurNAc, with this effect being more profound in the exponential growth phase. Scale bar, 10 μm.

Confirming the previously reported MurNAc auxotrophy of *T. forsythia* (strains OMZ 408, FDC 331, and the ATCC 43037 type strain) (Wyss, 1989), in this study, we visualized the effect of step-wise MurNAc depletion of the culture medium on *T. forsythia* ATCC 43037 cell morphology by applying SEM. In parallel experiments, we compared the situation in *T. forsythia* WT and *ΔTf_murK::erm* cells, in both the exponential and stationary growth phase (**Figure 6**).

For optimal growth of *T. forsythia*, MurNAc supplementation of the medium was routinely done at a concentration of 20 μg/ml, which in our previous experiment resulted in almost identical growth characteristics for the WT and *ΔTf_murK::erm* mutant, in both growth phases analyzed (**Figure 4**). Under this “optimal” condition, also no difference of either cell shape or cell aggregation was apparent from the SEM micrographs (**Figure 6**, lane 1).

However, we found that reduction of MurNAc supplementation, besides causing growth defects in *T. forsythia* cells, reflected by reduced cell densities at OD_600_ (cf. **Figure 4**), caused severe morphological changes, such as cell thickening and development of fusiform cell morphology (**Figure 6**). These morphological changes occasionally appeared in the *T. forsythia* WT already when the MurNAc concentration was reduced to 1 μg/ml (which is the second highest MurNAc concentration that was tested in the course of this study), but they became more evident when MurNAc in the medium was reduced to 0.5 μg/ml, and were dominant at 0.1 μg/ml, where almost exclusively fusiform cells were present (**Figure 6**). Whether the evident loss of aggregation capability of fusiform *T. forsythia* cells is a direct effect of the morphological change upon MurNAc depletion or rather of the concomitant decrease of overall cell density needs to be further investigated.

Intriguingly, *ΔTf_murK::erm* mutant cells appeared to be more tolerant toward MurNAc depletion compared to the *T. forsythia* WT implicating that they seemed to require less MurNAc to maintain their rod-shaped cells in comparison to the WT. Exponential-phase mutant cells grew mostly as normal rods in medium containing 1 or 0.5 μg/ml MurNAc. Only further reduction to 0.1 μg/ml MurNAc caused the transformation to fusiform morphology. In mutant cells from the stationary phase, fusiform cells were already visible at 0.5 μg/ml MurNAc and were prominent at 0.1 μg/ml MurNAc. Thus, morphological alteration of *T. forsythia* cells due to MurNAc depletion occurs in WT cells already at higher MurNAc concentration than in a *ΔTf_murK::erm* mutant.

## Discussion

In this study, a specific MurNAc-kinase of *T. forsythia* ATCC 43037, Tf_MurK (Tanf_08380), was biochemically characterized. Of all tested sugars only MurNAc and, albeit marginally, GlcNAc were phosphorylated by the kinase. The reaction of rTf_MurK with MurNAc as the sugar substrate was about 100-times faster than with the GlcNAc substrate (*V*_max_ of 52.6 *versus* 0.5 μmol/min mg). Moreover, with GlcNAc, rTf_MurK did not reach substrate saturation, reflected by an exceedingly high *K*_M_ of 116 mM. To clearly define substrate specificity of an enzyme acting on alternative substrates, the specificity constants (*k*_cat_/*K*_M_) have to be determined (Eisenthal et al., 2007). For Tf_MurK acting on MurNAc and GlcNAc, we determined *k*_cat_/*K*_M_ values of 52550 and 0.86 s^-1^ M^-1^, respectively, resulting in a ratio of the specificity constants of >60000, which demonstrates the enzyme’s strong preference for MurNAc. Moreover, the reaction product was identified as MurNAc-6P through cleavage by the specific MurNAc-6P etherase Tf_MurQ identified previously (Ruscitto et al., 2016). Thus, Tf_MurK can be unambiguously assigned as a specific MurNAc 6-kinase. The GlcNAc kinase activity of the enzymes is only marginal and likely physiologically not relevant.

This is the first report on an enzyme with a stringent substrate specificity for MurNAc. In 2011, Reith et al. reported a kinase from *Clostridium acetobutylicum* (Ca_MurK) with specificity for MurNAc (Reith et al., 2011). Although this enzyme was named MurK, too, it has only limited overall amino acid sequence identity with Tf_MurK (26.7%, *E*-value of 8.9 × 10^-4^) and was shown to act on both, MurNAc and GlcNAc, with a slight preference for the latter (ratio of specificity constants < 0.4-fold, and *k*_cat_/*K*_M_ = 225000 and 510000 s^-1^ M^-1^, for MurNAc and GlcNAc, respectively). The *K*_M_ value of Tf_MurK for MurNAc (200 μM) and *K*_M_ values of Ca_MurK for MurNAc (190 μM) and GlcNAc (127 μM) were in the same range, reflecting similar affinities of the enzymes for their substrates. Tf_MurK is slightly slower (turnover number *k*_cat_ = 10.5 s^-1^) compared to Ca_MurK (*k*_cat_ = 42.8 s^-1^ and 65 s^-1^, for MurNAc and GlcNAc, respectively), in agreement with its stringent substrate specificity.

Evaluation of the kinetic data indicated that Tf_MurK is inhibited by its substrate MurNAc, in excess. Substrate inhibition is a widespread phenomenon among enzymes (Reed et al., 2010; Yoshino and Murakami, 2015), leading to velocity curves that rise to a maximum and then descend as the substrate concentration increases. For some enzymes, substrate inhibition is a means of allosteric feedback regulation (Reed et al., 2010). The biological significance of the inhibition of Tf_MurK by MurNAc, however, is questionable, since the effect occurs only at very high MurNAc concentration (*K_i[S]_* of 4.2 mM).

Based on amino acid sequence identity Tf_MurK can be classified as a member of the BcrAD/BadFG-like ATPase family (PF01869). These kinases are proposed to require Mg^2+^ ions for ATP binding and catalytic activity. Mg^2+^-dependency of Tf_MurK was confirmed in this study. Tf_MurK was found to be rather unstable. The enzyme loses activity within minutes at temperatures ≥ 20° C, which may explain problems with obtaining catalytically active pure enzyme in a previous study (Ruscitto et al., 2016). MurNAc 6-kinase activity of Tf_MurK, was indirectly shown in that study by rescuing growth on MurNAc of an *E. coli* mutant defective in the MurNAc-specific phosphotransferase type transporter Ec_MurP by providing a plasmid expressing both Tf_MurT and Tf_MurK, but not by expressing one of the two proteins alone (Ruscitto et al., 2016). Rapid degradation of rTf_MurK might also be the reason for the appearance of smaller bands besides the major protein band in SDS-PAGE analysis (**Figure 1**). A crystal structure of a Tf_MurK-like protein of *Porphyromonas gingivalis* (PG1100) deposited to the structure database by the Northeastern Structural Genomics Consortium (pdb code 1ZBS) revealed an open and flexible structure that might explain the functional instability.

Wyss (1989) reported that *T. forsythia* strains OMZ 408, FDC 331 and ATCC 43047 strictly depend on MurNAc for growth and rod-shaped cell morphology. According to that study, a *T. forsythia* cell population remained morphologically homogeneous and cell densities exceeded 10^7^ cells per ml, when grown in a medium supplemented with 1 μg/ml of MurNAc, whereas 0.1 μg/ml MurNAc was found to be the minimal effective concentration, with a large portion of cells developing into unusual spherical or spindle-formed “fusiform” cells (Wyss, 1989). Growth experiments presented in the course of this study confirmed MurNAc auxotrophy for *T. forsythia* ATCC 43037. Moreover, we imaged morphological changes of *T. forsythia* cells in response to MurNAc limitation by SEM. We observed an evident growth defect of *T. forsythia* WT cells already with 1 μg/ml of MurNAc in the medium (**Figure 4**) and, consistently, the cells grown under these conditions were shown to exhibit severe morphological alterations, starting with thickening and shortening of the cells, followed by converting to fusiform cell-shape, and finally appearance of thick “lemon-shaped” cells (**Figure 6**). Surprisingly, the *ΔTf_murK::erm* mutant was less affected by MurNAc limitation than the *T. forsythia* WT. This was evident already from the recorded growth curves (**Figure 4**), and supported by less pronounced morphological changes upon growth in MurNAc-limited medium (**Figure 6**).

**FIGURE 7:**
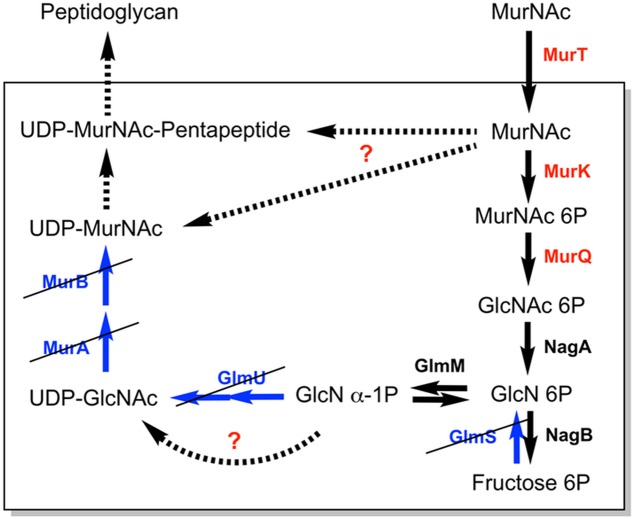
Simplified scheme of the MurNAc and PGN metabolic pathways in *T. forsythia*. Proteins required for MurNAc uptake and utilization, encoded by the *Tf_murTKQ* operon of *T. forsythia,* are shown in red. Some generally essential and well-conserved bacterial PGN biosynthetic enzymes are missing in *T. forsythia* (colored in blue, crossed out). Black arrows indicate metabolic enzymes (solid) and pathways (dashed) that are apparently present in *T. forsythia*. Question marks indicate enzymes or pathways that are suggested to be present in *T. forsythia* based on results obtained in the frame of this study.

The accumulation of MurNAc in *ΔTf_murK::erm* mutant cells confirms the function of Tf_MurK as a MurNAc kinase and demonstrates effective MurNAc uptake but a lack of catabolization capability of MurNAc caused by deletion of Tf_MurK. In WT cells, in contrast, MurNAc does not accumulate; it is catabolized via Tf_MurK and the downstream acting Tf_MurQ enzyme (**Figure 7**). Recently, a MurNAc transporter of the major facilitator superfamily (Tf_MurT) was identified in *T. forsythia* which is highly conserved within the *Bacteroidetes* phylum of bacteria (Ruscitto et al., 2016). Our data strongly suggest that the bacterium salvages MurNAc from the medium using this transporter and mainly channels it to the catabolic pathway. Besides utilizing MurNAc and presumably also muropeptides (Ruscitto et al., 2017) as nutrient source, *T. forsythia* assumedly also salvages these compounds directly for cell wall biosynthesis. Since *T. forsythia* is auxotrophic for MurNAc but a mutant defective in this kinase is still viable, an additional pathway must exist in the pathogen that shunts MurNAc to the peptidoglycan biosynthesis. Accumulation of the PGN precursor UDP-MurNAc-pentapeptide in stationary phase *ΔTf_murK::erm* cells shows that part of the MurNAc is indeed used for cell wall synthesis. In exponential phase cells, however, the levels of UDP-MurNAc-pentapeptide are low, as this precursor is readily consumed for peptidoglycan biosynthesis during bacterial growth. In stationary phase, when PGN biosynthesis is slowed down, UDP-MurNAc-pentapeptide levels increase dramatically, since the membrane-located steps of PGN biosynthesis are rate limiting (Lara et al., 2005). We have recently identified a salvage pathway for MurNAc that bypasses *de novo* biosynthesis of the PGN precursor UDP-MurNAc (Gisin et al., 2013; Borisova et al., 2014, 2017; Renner-Schneck et al., 2015). This pathway presumably is present in many Gram-negative bacteria, including *T. forsythia* and other members of the Bacteroidetes phylum. Interference with this pathway in *Pseudomonas* sp. leads to increased susceptibility to the antibiotic fosfomycin in pathogens harboring the target enzyme MurA (Gisin et al., 2013; Borisova et al., 2014). However, as *T. forsythia* lacks MurA, inhibition of the MurNAc salvage route will likely block PGN biosynthesis in this organism. We are currently attempting to characterize the route from MurNAc to the PGN biosynthesis, which represents a valuable target for the treatment of *T. forsythia*-associated periodontal diseases (**Figure 7**).

## Conclusion

A new sugar kinase from the oral pathogen *T. forsythia* was characterized, showing narrow specificity for MurNAc and an essential role in MurNAc catabolism in this organism. Surprisingly, the kinase mutant revealed a growth benefit and less morphological perturbations under MurNAc limitation conditions, indicating that a block in MurNAc catabolism affects peptidoglycan biosynthesis. The detailed characterization of the MurNAc kinase Tf_MurK and the *ΔTf_murK::erm* mutant increases our understanding of the unique cell wall and amino sugar metabolism of the oral pathogen *T. forsythia* that may pave new routes for lead finding in the treatment of periodontitis.

## Author Contributions

IH cloned rTf_MurK, biochemically characterized the enzyme, and conducted kinetic experiments by coupled enzymatic and HPLC-MS assays. IH analyzed the accumulation of metabolites by HPLC-MS. MT constructed and confirmed the *ΔTf_murK::erm* mutant. VM and VF conducted growth experiments with *T. forsythia* strains and VM conducted the SEM experiments with help of VF and prepared cells for accumulation studies. MC and AT synthesized substrates for the kinetic studies. CS and CM formulated the original problem and provided guidance troughout the study. IH and CM designed the experiments and developed methodology. IH, CS, and CM wrote the manuscript. CM resolved final approval of the version to be published.

## Conflict of Interest Statement

The authors declare that the research was conducted in the absence of any commercial or financial relationships that could be construed as a potential conflict of interest.
