# Supplementary material for: N-Acetylmuramic Acid (MurNAc) Auxotrophy of the Oral Pathogen Tannerella forsythia: Characterization of a MurNAc Kinase and Analysis of Its Role in Cell Wall Metabolism
Source: Front Microbiol. 2018 Jan 26;9:19. doi: 10.3389/fmicb.2018.00019 (PMC5790795; doi:10.3389/fmicb.2018.00019)
Supplement: Supplementary file 1 [file Data_Sheet_1.DOCX]

Supplementary Material

***N*-Acetylmuramic acid (MurNAc) Auxotrophy of the Oral Pathogen *Tannerella forsythia*: Characterization of a MurNAc Kinase and Analysis of its Role in Cell Wall Metabolism**

***Isabel Hottmann^1^, Valentina M. T. Mayer^2^, Markus B. Tomek^2^, Valentin Friedrich^2^, Matthew B. Calvert^3,4,5^, Alexander Titz^3,4,5^, Christina Schäffer^2*^ and Christoph Mayer^1*^***

*** Correspondence:** [christina.schaeffer@boku.ac.at](mailto:christina.schaeffer@boku.ac.at), , [christoph.mayer@uni-tuebingen.de](mailto:christoph.mayer@uni-tuebingen.de),

# Supplementary Figures and Tables

## Supplementary Figures S1-S5


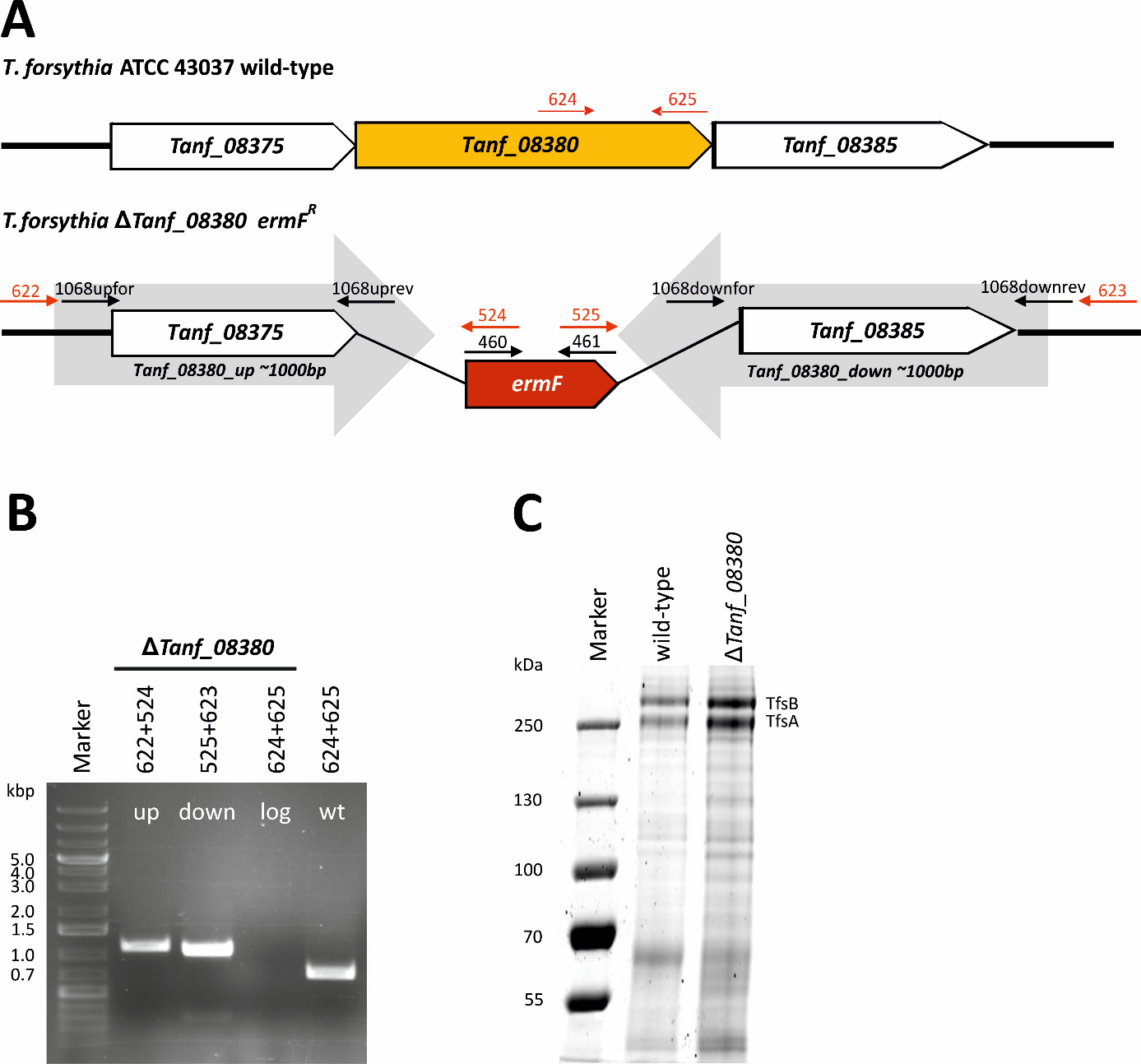


**FIGURE S1 |**Strategy for the generation of a *T. forsythia* ATCC 43037 *murK* deficient mutant and confirmation by PCR. **(A)** The genomic organization of the *Tanf_08380* locus is shown for the parent strain *T. forsythia* ATCC 43037 and the Δ*murK* (*Tanf_08380***)** mutant. Black colored arrows represent primers used for PCR amplification of genes and homologous regions, red colored primers represent those used to screen for correct integration of the knock-out (not drawn to scale). **(B)** Agarose gel electrophoresis confirms the deletion of *Tanf_08380* using the up-stream primers 622/524 (1113 bp) and down-stream primers 525/623 (1045 bp) on genomic DNA of *T. forsythia* ATCC 43037 Δ*Tanf_08380* mutant with integrated *ermF*. Primers 624/625 yield in a 700-bp PCR fragment when using *T. forsythia* wild-type genomic DNA, whereas this fragment is absent on genomic DNA of the Δ*Tanf_08380* mutant confirming the loss of the gene (log); O´Gene Ruler 1‑kb Plus DNA Ladder (Thermo Fisher Scientific) was used as a gene ladder. **(C)** A Coomassie Brilliant Blue-stained SDS-PAGE gel of crude cell extracts from *T. forsythia* WT and Δ*murK* after separation on a 7.5% SDS-polyacrylamide gel. The high-molecular weight glycoproteins TfsA and TfsB are indicated. PageRuler Prestained Protein Ladder (Thermo Fisher Scientific) was used as a molecular weight marker.

**
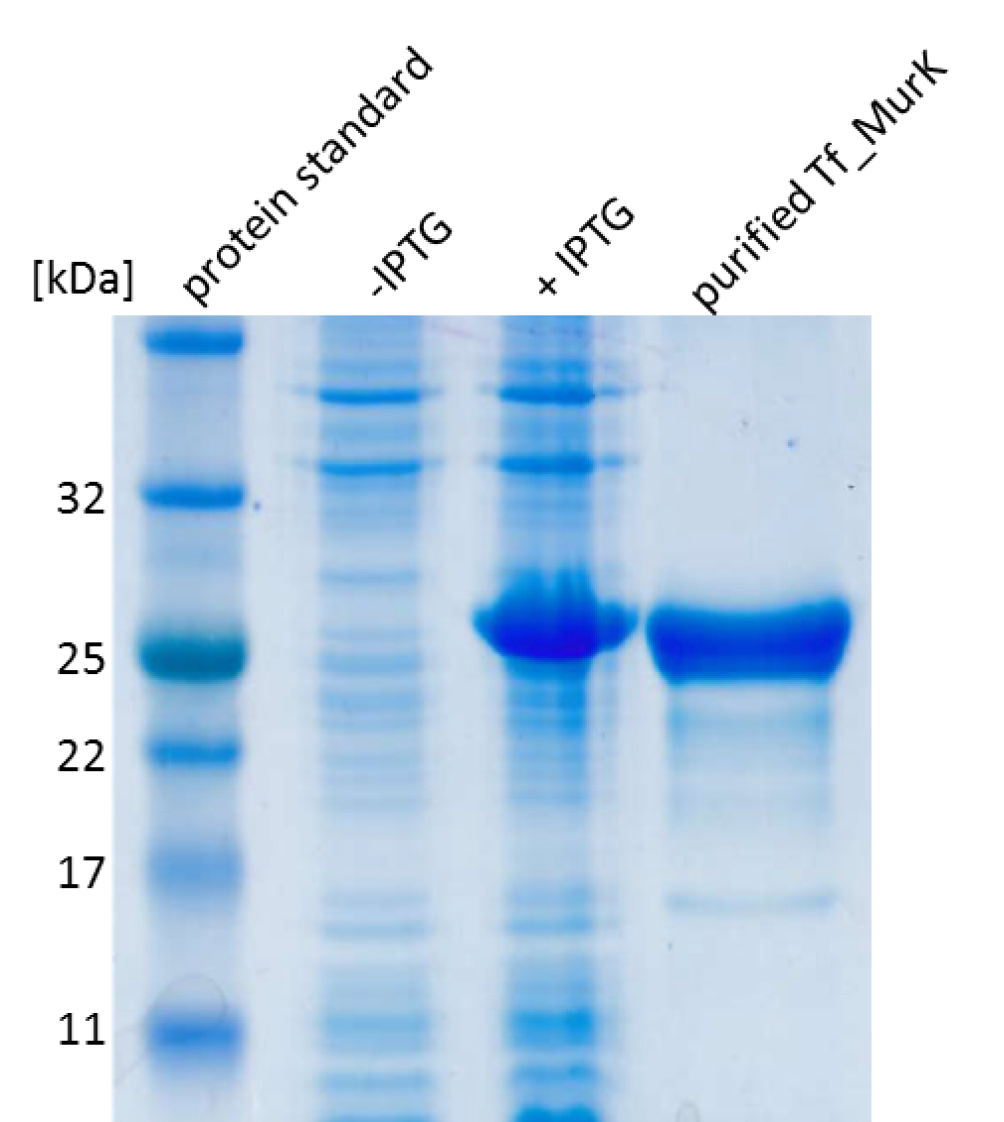
**

**FIGURE S2 | Purity of Tf_MurK.** Expression and purity of rTf_MurK as analyzed by SDS-PAGE using CBB staining. Lane 1, protein size standard mixture; lane 2, *E. coli* cell extract before (-IPTG) and, lane 3, after (+IPTG) induction; lane 4, 10 µg of rTf_MurK protein purified by Ni^2+^ affinity chromatography and gel filtration.





**FIGURE S3 | Temperature and pH stability and optima of rTf_MurK**. The effect of different pH (left) and temperature (right) on the rTf_MurK activity is shown; optimum (blue) and stability (red). Data were determined in triplicates as described in materials and methods. Standard errors (SEM) are indicated and calculated out of three biological replicates.

**

**

**FIGURE S4 | Tf_MurK kinetic parameters.** Kinetic parameters were determined using the coupled enzyme ATPase assay as described in Material and Methods. Shown are the hyperbolic curves according to Michaelis-Menten (black) and a fitting considering substrate inhibition (blue). *T. forsythia* MurK showed higher *v_max_* and a lower *K*_m_ for MurNAc (left) compared to GlcNAc (right).





**FIGURE S5 | Quantification of accumulation products by ESI-TOF-MS analysis.** Shown are the extracted ion chromatograms (EIC) for the double charged ion of UDP-MurNAc-pentapeptide ((M-2H)^2-^ =595.670)) in grey and for MurNAc (M-H)^-^ =292.110) in black. In *T. forsythia* WT only UDP-MurNAc-pentapetide accumulates in **(A)** exponential and in **(B)** stationary phase. In *∆Tf_murK::erm* cells primarily MurNAc and also UDP-MurNAc-pentapeptide accumulate in **(C)** exponential phase. Accumulation ratio changes in **(D)** stationary phase in *∆Tf_murK::erm* cells. To determine the relative amount of accumulation products the EICs of UDP-MurNAc-pentapeptide and MurNAc were analyzed using Graph Pad Prism6 to calculate the area under the curve (see **Figure 5**).

**
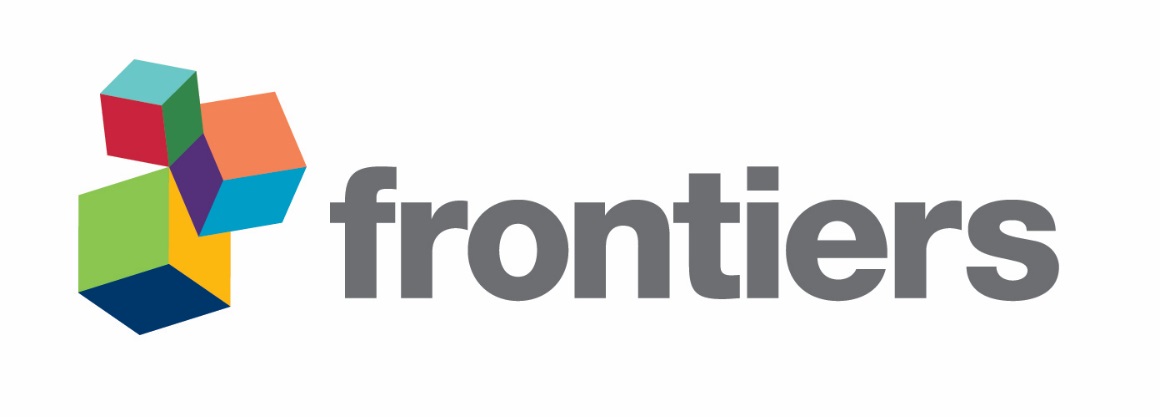
**
